# “College fields of study and substance use”

**DOI:** 10.1186/s12889-020-09722-1

**Published:** 2020-10-30

**Authors:** Wei-Lin Chen, Jen-Hao Chen

**Affiliations:** 1 Center for Teacher Education, National Sun Yat-sen University, Kaohsiung City, Taiwan; 2 Department of Sociology and Department of Psychology, National Chengchi University, 64, Section 2, Zhinan Rd., Taipei City, Taiwan

**Keywords:** Education, College major, Health behaviors, Health disparities, Substance use

## Abstract

**Background:**

Numerous studies have documented factors that are associated with substance use behaviors among college-aged individuals. However, relatively few studies have considered the heterogeneity of the college experience by field of study (i.e., college major) and how that educational context might affect students’ health behaviors differently. Drawing from theories and prior research, this study investigates whether college majors are associated with different substance use behaviors, both during college and upon graduation.

**Methods:**

The study analyzed longitudinal data from the National Longitudinal Survey of Youth 97 (*N* = 1031), specifically data on individuals who obtained a bachelor’s degree, to examine the associations between college fields of study and trajectories of three substance use behaviors: smoking, heavy alcohol use, and marijuana use.

**Results:**

The results indicate that social science and business majors were associated with more substance use behaviors than arts and humanities and STEM majors. However, social science majors were associated with a faster decrease in substance use behaviors over time. Importantly, the differences we found in mean levels of substance use behaviors and trajectories were not explained by demographic characteristics, family SES background, childhood health conditions, and employment experience. Further analysis that examined college major and each substance use behavior individually suggests that the associations were stronger for heavy alcohol use and marijuana use. Moreover, we found the associations were more pronounced in men than women.

**Conclusions:**

The study finds that not all college majors show the same level of engagement in substance use behaviors over time, and that the associations also vary by (1) the specific substance use behavior examined and (2) by gender. These findings suggest it is important to consider that the different learning and educational contexts that college majors provide may also be more or less supportive of certain health behaviors, such as substance use. Practical implications are discussed.

## Introduction

Substance use is an important public health concern in the United States. National surveys consistently show that substance use peaks during emerging adulthood [1]. Although college students may show less substance use than non-students in the same age range [2], it remains true that smoking, heavy alcohol use, and illicit drug use are not uncommon [3–5] among college students and are considered pressing health issues [6]. O’Mally and Johnston’s [7] influential study shows a high prevalence of heavy alcohol use and smoking among college students, with only a slight improvement from 1980 to late 1990. Even the most recent national survey data suggest that substance use remains a pressing health concern of the college-age population. The national Monitoring the Future 2018 survey indicates that among full-time college students in the United States, 15.3% have used cigarette, 29% are heavy alcohol users, and 24.7% have used marijuana during the past 30 days [4].

Moreover, research makes clear that substance use during the college years has significant consequences for learning and health. College students who are heavy alcohol users are more likely to get injured [8], have lower academic performance and drop out of college at higher rates [9], and demonstrate poor working memory [10]. Marijuana can impair neuropsychological functioning and thus affect individuals’ learning and work performance [11]. Smoking is associated with lower cognitive function among college students, including a lower level of verbal or auditory competence [12]. Because so many college students use substances and their negative impact on physical health and learning can be significant, it is critical to investigate and understand the factors that relate to students’ substance use behaviors.

There are many prior studies that contribute to our understanding of the risk and protective factors that may promote or deter substance use among college students [13–16]. While a full review of the extant studies of substance use among college studies is beyond the scope of this research, it is useful to briefly summarize factors that have been shown to relate to college students’ substance use. Furthermore, studies using large-samples suggest that substance use behaviors (such as heavy alcohol use, smoking, marijuana use) among college students tend to co-occur [17, 18], suggesting the need to investigate substance use behaviors simultaneously. Following Ham and Hope’s [19] approach in their influential systematic review of problematic drinking among college students, we classify previously identified risk and protective factors of substance use at three levels: individual, interpersonal, and contextual. First, substance use varies by individual demographic characteristics and personality factors. For example, studies consistently find that men have a higher likelihood of substance use than women [13, 20] and that African American and Hispanic students have lower rates of substance use [13, 20, 21] than white students. Other studies find that certain personality traits appear to be associated with substance use among college students. For example, sensation seeking is related to heavy alcohol use [22, 23].

However, individual factors offer little help in prevention and intervention. Therefore, in recent years, research has moved to investigate the role of interpersonal and contextual factors on college students’ substance use. For example, living with parents during college is associated with lower levels of substance use [2]. In contrast, two systematic literature reviews of problematic drinking and smoking suggest that living on campus appear to correlate with increased alcohol consumption and smoking [24, 25]. In contrast, low academic performance, often measured by GPA, is associated with alcohol or illicit drug use. Heavy alcohol or drug use may impact cognitive functioning, which contributes to poorer grades. Evidence also shows that working part-time during college is associated with more substance use [26, 27]. Membership in fraternities and sororities is found to be associated with substance use [28, 29]. Finally, several studies start to pay attention to the educational context. In a systematic literature review by Carter and colleagues [25], they conclude that full-time college students, especially for those in 4-year college, display a greater engagement in heavy alcohol use. Cranford and colleagues [30] analyze a probability sample of students and find that undergraduates are associated with a higher likelihood of heavy alcohol use and marijuana use (but not smoking) than graduate students.

Although prior studies have investigated a wide range of individual, interpersonal, and contextual factors that relate to substance use among college students, the role of college major has received relatively little attention. This is a curious oversight because college education, by nature, is more heterogeneous than secondary education. Even within the same college, majors vary on curricula, expectations, learning environment, and level of professionalization. In addition, majors differ on whether and how much they expect students to learn specialized knowledge, gain hands-on experience, and collaborate on group projects [31, 32]. Because a student’s academic experience differs so much by major and is so central to life during the college years, it is reasonable to believe that college major may affect students’ likelihood of engaging in substance use behaviors as they emerge into adulthood. In other words, some of the differences that exist across majors may be more or less protective against, or supportive of, students’ substance use. This study aims to addresses this key, relatively unexplored question: Does a student’s college major predict his/her likelihood of substance use during and after college?

Based on prior studies, there are strong empirical and theoretical reasons to believe that engaging in a health risk behavior, such as substance use, may vary by college major. First, only some majors expose students to knowledge of human health and physiology, which may produce differences in health literacy by major [33]. Differences in health literacy may, in turn, lead to differences in health behaviors. Second, the undergraduate socialization model conceptualizes college as the primary socialization field for young adults’ development [34, 35]. Students are socialized into the norms of their major and participate in activities and social interactions that promote their success in related professional fields. Social learning theory posits that individuals learn from various forms of interaction with peers and colleagues, which highlights the importance of how students’ interactions in their major may affect how they learn health behaviors, such as substance use [36–38]. For example, health-related majors may be trained to avoid smoking and drug use because they will likely work in smoke-free and drug-free workplaces when they graduate. In contrast, business majors might be socialized to be more tolerant toward smoking and heavy alcohol use because those behaviors occur in the social interactions that graduates have with their clients. In these and other ways, college majors provide a different environment and socialization that may affect health behaviors.

Despite these reasons to believe that a student’s choice of college major may affect their substance use, there is limited empirical evidence on this research question. Of the studies that do exist on the substance use behaviors of college students, many rely on surveys at a single college or university (e.g.), [39–41]. Even fewer studies exist that consider college major as an influential factor in substance use behaviors over time. Finally, to our knowledge, it appears that no study exists that examines this question with the benefit of a large-scale, national sample with longitudinal data. This study aims to address these limitations by using a large-scale, longitudinal dataset to investigate whether and how engagement in substance use behaviors (i.e., smoking, heavy alcohol use, and marijuana use) varies by college major.

## Methods

### National Longitudinal Survey of youth 1997

This study used data from the National Longitudinal Survey of Youth 1997 (NLSY97), a nationally representative sample of youths who were born between 1980 and 1984. The NLSY97 began by interviewing 8984 respondents who were 12 to 18 years old in 1997–1998 (round 1). Respondents were followed every year until 2013–2014 (round 16). After that, respondents were followed every 2 years [42]. The NLSY97 aims to understand U.S. youths’ transition from school to work and into adulthood [43]. The NLSY97’s detailed information on college education, together with the large-scale, longitudinal national sample, provide a rare opportunity to examine college majors and substance use over time.

We used transcript data in the NLSY97 to identify when a respondent started college and when s/he received a college degree. College transcripts provide the most accurate information on when individuals started college and whether they received a bachelor’s degree. We limited the study to respondents who obtained a bachelor’s degree between 2001 and 2011 because no college transcript data was collected after 2011. After excluding any individual whose college major could not be identified or was missing, we were left with a sample of 1099 youths who completed college, obtained their degree between 2001 and 2011, and whose college major was known. A small proportion of youth in our sample had missing values on the variables of interest for health behaviors (i.e., smoking, heavy alcohol use, and marijuana use). For each youth, three rounds of data were used in the analysis: the wave when the respondent entered college, the wave when the respondent finished college, and the wave after college completion. The final sample for longitudinal analysis included 1031 youth.

### Classification of college major

In each round of data collection in the NLSY97, respondents who enrolled in college were asked about their major(s); we used this self-reported information to identify the field of the college degree. We chose to rely on self-reports, rather than college transcript data, to identify college majors because the college transcript data from the NLSY97 did not provide raw data on specific majors. Instead, the NLSY97 raw data grouped college majors into categories, such as liberal arts, sciences, general studies, and humanities. Thus, it was unclear what a student’s exact college major was or how the NLSY97 defined its college major categories. Second, we chose to structure our data with self-reported college major rather than NLSY97-defined categories because it would allow future researchers to reclassify majors according to different definitions and research needs. We matched self-reported college major to the college transcript data for the year when respondents received a bachelor’s degree.

We followed the National Science Foundation’s (NSF) classification to group college majors into eight categories [44]. If a major appeared that was not on the NSF list, we followed the definitions of Liu, Sun, & Winters [45] because they expanded the NSF classification to include as many majors as possible in their study. If a respondent reported more than one major and the two majors were in the same field, we placed that respondent in the corresponding category (e.g., social sciences or STEM). If a respondent reported more than one major and the two majors were in different fields, we placed the respondent in the “multiple fields” category. Detailed definitions of each group are presented in Table 1.

**Table 1:** Classification of College Fields of Study in the NLSY97 Dataset

**STEM**	
physical sciences, agriculture sciences, biological and biomedical sciences, computer/information sciences, communication technologies, engineering technologies, science technologies, military technologies and applied sciences, engineering, mathematics, statistics	
**Health**	
nursing, health professions, pre-dental, pre-med, pre-vet, residency programs	
**Education**	
education, high-school/secondary programs	
**Arts & Humanities**	
architecture/environmental design, area studies, communications, journalism, English, ethnic studies, fine and applied arts, foreign languages, history, interdisciplinary studies, philosophy, theology/religion studies, personal and culinary services, general studies and humanities, precision production, visual and performing arts	
**Social Sciences**	
anthropology, archaeology, criminology, home economics, political science and government, psychology, sociology, pre-law, family and consumer sciences, human sciences, legal professions, homeland security, law enforcement, firefighting and related protective services, public administration and social service professions, social sciences,	
**Business**	
business management, economics, marketing	
**Others**	
agriculture/natural resources, un-codable, construction trades, mechanic and repair technologies, transportation and materials moving	
**Multiple Fields**	
individuals who have more than one major when the majors are from different fields	

### Measures of substance use behaviors

The NLSY97 asked respondents to report substance use behaviors during the past month for smoking, healthy alcohol use, and marijuana use. The questions were: “During the past 30 days, on how many days did you smoke a cigarette?”; “On how many days did you have five or more drinks on the same occasion during the past 30 days?”; and “On how many days have you used marijuana in the last 30 days?” We first converted respondents’ answers to binary variables, coding a response ‘one’ if a respondent’s answer indicated his or her engagement of the substance use behavior during the past 30 days, and ‘zero’ if otherwise. Next, because substance use behaviors sometimes cluster together, we generated a new variable for ‘degree of engagement in substance use behaviors.’ For this variable, we summed the three binary indicators to create an indicator of numbers of substance use behaviors (ranging from 0 to 3) in the past month.

### Covariates

The statistical analysis in this study controlled for potential confounders of substance use behaviors. The confounders included race/ethnicity, gender, highest level of parental education, age, childhood health problems (i.e., physical conditions, learning problems, and other chronic conditions), years of degree completion, self-rated general health, number of weeks working at a job during the year, and an income-to-poverty ratio for each year. In the baseline survey, parents were asked if the respondent had the following three childhood health problems that may potentially limit school in early life: (1) a physical condition, (2) a learning problem, and (3) any chronic condition.

### Empirical strategy

The first step in the empirical analysis was to use negative binomial regressions to estimate whether receiving a bachelor’s degree was associated with degree of engagement in substance use behaviors in 2011. After completing this cross-sectional analysis, we applied multilevel negative binomial models to estimate the associations between college major and degree of engagement in substance use behaviors over time. We used the multilevel model approach to account for the nested nature of individual longitudinal data because one’s substance use at different time periods is nested within the invariant characteristics of the person [46]. Specifically, in our statistical model, level 1 represents individual substance use behaviors over time and level 2 represents personal characteristics. Furthermore, we used negative binomial regression in conjunction with the multilevel model approach. The negative binomial regression is widely used to model count data [47] and has been widely applied in substance use research (e.g.), [48–50]. More specifically, negative binomial regression can be used for over-dispersed count data, that is when the conditional variance exceeds the conditional mean. As such, it can be considered as a more generalized version of Poisson regression and becomes more efficient than Poisson when the outcome variable is over-dispersed [47]. In the longitudinal analysis, we also included interaction terms between college major and age to investigate whether substance use behaviors change over time by different majors. All regressions controlled for potential confounders.

For all regressions, we selected arts & humanities majors as the reference group. In addition, we did pair-wise comparisons across college majors using the same model and controlling for the full set of confounders. As such, we were able to fully test whether the association was statistically significant between each major. All analyses were done using Stata 16. This study is exempt from IRB review because it uses a survey dataset that is publicly available.

## Results

Table 2 presents sample characteristics for all respondents who obtained a bachelor’s degree between 2001 and 2011, as well as respondents’ substance use behaviors at the time of entering college and at the time of graduation, by college major. At the time of entering college, we observe substantial variations in substance use behavior by college major. For example, students who major in STEM, health sciences, and education showed a lower degree of engagement in substance use behaviors. In contrast, students who major in business, on average, showed a higher degree of engagement in substance use behaviors. A closer look at Table 2 also reveals a trend over time of increased substance use behaviors for individuals in nearly all majors. In other words, for students in nearly all majors, substance use at graduation was more prevalent than it was at college entry.

**Table 2:** Descriptive Statistics of the NLSY 97 Sample at College Entrance Year and College Completion Year for the Full Sample and By College Fields of Study (*N =* 1031)

	STEM	Health	Education	Arts & Humanities	Social Sciences	Business	Others	Multiple	Full Sample
***Health Behaviors***									
**College Entrance**									
Substance Use Behaviors (0–3)	0.63 (0.89)	0.63 (0.91)	0.61 (0.93)	0.65 (0.95)	0.71 (0.97)	0.84 (1.02)	0.71 (1.05)	0.77 (0.96)	0.71 (0.96)
**College Graduation**									
Substance Use Behaviors (0–3)	0.77 (0.94)	0.64 (0.82)	0.64 (0.73)	0.84 (0.98)	0.75 (0.98)	0.92 (1.01)	0.68 (0.82)	0.85 (0.93)	0.80 (0.95)
**Baseline (College Entrance)**									
***Demographics***									
Race/Ethnicity									
White (%)	75.88	68.75	69.88	69.09	54.97	65.20	74.29	74.36	67.94
African American (%)	8.82	10.42	13.25	15.00	20.47	15.20	17.14	15.38	14.69
Hispanic (%)	9.41	12.50	15.66	9.55	18.71	13.24	2.86	7.69	11.93
Asian (%)	3.53	0.00	1.20	3.18	2.92	4.41	5.71	2.56	3.15
Other and missing (%)	2.35	8.33	0.00	3.18	2.92	1.96	0.00	0.00	2.29
Gender									
Male (%)	62.35	12.5	15.66	41.36	32.16	55.39	45.71	31.62	41.7
Female (%)	37.65	87.5	84.34	58.64	67.84	44.61	54.29	68.38	58.3
Parental Education									
BA & more (%)	62.35	58.33	42.17	60.91	43.86	47.06	42.86	59.83	53.34
HS (%)	17.65	12.5	31.33	13.18	30.41	18.63	22.86	23.08	20.61
Some College (%)	17.06	27.08	25.3	22.73	23.98	29.41	28.57	13.68	22.9
Missing (%)	2.94	2.08	1.20	3.18	1.75	4.90	5.71	3.42	3.15
***Baseline Mental Health Problem***									
Physical Condition (%)	2.94	6.25	2.41	5.91	4.09	2.45	0.00	5.98	4.01
Learning Problem (%)	1.76	2.08	0.00	4.09	5.26	0.98	0.00	5.98	2.96
Chronic Condition (%)	10.00	4.17	7.23	10.45	11.70	8.82	8.57	8.55	9.45
Age	18.34	18.69	18.46	18.51	18.60	18.65	18.23	18.30	18.50
	(0.83)	(1.32)	(1.00)	(1.12)	(1.22)	(1.27)	(1.03)	(0.71)	(1.09)
Self-rated General Health	4.27	4.25	4.18	4.20	4.11	4.26	4.06	4.23	4.21
	(0.78)	(0.67)	(0.78)	(0.86)	(0.81)	(0.78)	(0.84)	(0.80)	(0.80)
Income to Poverty Ratio	469.21	498.86	395.46	521.85	453.18	539.44	447.63	490.23	487.09
	(440.48)	(266.44)	(208.72)	(475.96)	(534.75)	(569.75)	(467.69)	(473.38)	(475.41)
Weeks of Working at Employed Job	587.11	647.53	752.44	591.29	675.95	759.88	689.73	711.61	668.91
	(527.36)	(570.88)	(551.99)	(544.68)	(585.82)	(704.09)	(506.45)	(618.59)	(594.14)
Years of Degree Completion	4.79	4.90	5.04	4.85	5.00	4.94	4.86	4.73	4.89
	(1.37)	(1.69)	(1.60)	(1.55)	(1.77)	(1.61)	(1.56)	(1.25)	(1.55)

Standard deviations are presented in parentheses

Table 3 presents the results of regression analyses that examined the association between level of education and substance use behaviors. The first column shows results for the full sample; the second and third columns show results for men and women separately. For the full sample, Model 1 found that individuals with a bachelor’s degree were less likely to engage in substance use behaviors, compared to individuals without a bachelor’s degree. Model 2 found that individuals with a STEM or education major were less likely to engage in substance use behaviors than individuals without a bachelor’s degree. While the coefficients of other majors were in the expected direction, they were not statistically significant. In addition, some gender differences were observed.

**Table 3:** Association Between Education and Substance Use Behaviors in 2011 from Negative Binominal Regression, Cross-Sectional Analyses

	Full Sample (*N* = 6949)				Men (*N* = 3461)				Women (*N* = 3488)			
	Coefficient	S.E.	95% C.I.		Coefficient	S.E.	95% C.I.		Coefficient	S.E.	95% C.I.	
***Model 1*** ***Ref: No College***												
College Degree	−0.167***	0.042	− 0.249	− 0.084	− 0.114*	0.057	− 0.226	− 0.002	− 0.237***	0.063	− 0.360	− 0.114
***Model 2*** ***Ref: No College***												
STEM	− 0.266**	0.095	− 0.453	− 0.080	− 0.254*	0.114	− 0.478	− 0.031	− 0.275	0.175	− 0.618	0.067
Health	− 0.197	0.191	− 0.571	0.176	− 0.001	0.379	− 0.745	0.743	− 0.298	0.221	− 0.731	0.135
Education	− 0.751***	0.190	−1.124	− 0.378	− 0.455	0.354	−1.149	0.240	− 0.882***	0.226	−1.324	− 0.439
Arts & Humanities	− 0.126	0.083	− 0.287	0.036	− 0.133	0.117	− 0.362	0.097	− 0.139	0.117	− 0.368	0.090
Social Sciences	−0.071	0.094	−0.256	0.114	0.005	0.141	−0.271	0.281	−0.139	0.127	−0.388	0.110
Business	−0.073	0.082	−0.234	0.088	0.034	0.099	−0.160	0.229	−0.272	0.147	−0.560	0.017
Others	−0.262	0.214	−0.682	0.158	−0.205	0.269	−0.731	0.322	−0.332	0.355	−1.027	0.363
Multiple Fields^a^	−0.091	0.114	−0.316	0.133	−0.243	0.202	−0.638	0.153	−0.043	0.140	−0.317	0.231

*** *p* < .001, **p < .01, *p < .05

Abbreviations: *Ref.*, reference, *S.E.* standard error, *C.I.* confidence interval

Model 1 and Model 2 include all covariates. All models include no college degrees

^a^Multiple Fields refers to individuals with a double major in different fields of college major only; individuals with a double major in the same field of college major are not categorized into Multiple Fields

Table 4 presents the results from the multilevel negative binomial models that estimated the associations between college major and substance use behaviors over time. Again, the first column shows results for the full sample and the second and third columns show results for men and women separately. Results from the full sample suggest that the main effect of college major showed some variation in substance use behaviors. In addition, interaction terms showed that changes in substance use behaviors with age also differed by major. For example, individuals who majored in social sciences and business (marginally significant) were associated with decreased substance use with age, compared to individuals in arts and humanities majors. Importantly, the results in this table also show that differences between these college majors in substance use and changes over time cannot be explained by the covariates, including demographic characteristics, family SES background, childhood health, and employment. We also performed additional statistical tests that did pair-wise comparisons across majors; these analyses indicated some interesting patterns (results are in the Appendix: Table 6). For example, individuals with a social science major were also less likely to engage in substance use over time than individuals in a STEM major.

**Table 4:** Longitudinal Results of the Association Between College Fields of Study and Substance Use Behaviors from Multilevel Negative Binominal Regression (*N =* 1031)

	Full Sample (*N* = 1031)				Men (*N* = 428)			Women (*N* = 603)				
	Coefficient	S.E.	95% C.I.		Coefficient	S.E.	95% C.I.		Coefficient	S.E.	95% C.I.	
***Major (Ref: Arts & Humanities)***												
STEM	0.138	0.673	−1.182	1.457	0.887	0.515	−0.122	1.896	−0.392	0.489	−1.350	0.567
Health	0.930	1.162	−1.349	3.208	−0.791	1.626	−3.977	2.396	0.531	0.602	−0.649	1.710
Education	0.945	0.925	−0.869	2.758	−0.352	0.951	−2.216	1.512	0.714	0.515	−0.295	1.724
Social Sciences	1.721*	0.672	0.403	3.039	2.233***	0.606	1.045	3.422	0.366	0.438	−0.493	1.224
Business	1.533*	0.747	0.069	2.998	1.721**	0.514	0.714	2.729	0.084	0.482	−0.861	1.030
Others	−0.045	1.311	−2.615	2.525	0.711	0.958	−1.167	2.589	−0.423	0.899	−2.186	1.340
Multiple Field	0.995	0.769	−0.513	2.503	1.605*	0.709	0.216	2.994	0.020	0.472	−0.905	0.945
***Age***	0.217***	0.049	0.120	0.313	0.285*	0.127	0.037	0.534	−0.017	0.103	−0.218	0.184
***Change in Substance Use by Major***												
STEM × Age	−0.014	0.030	−0.072	0.044	−0.048*	0.022	−0.092	−0.004	0.017	0.022	−0.026	0.060
Health × Age	−0.046	0.051	−0.146	0.054	0.023	0.071	−0.117	0.163	−0.026	0.026	−0.077	0.026
Education × Age	−0.046	0.041	−0.126	0.034	0.018	0.041	−0.062	0.097	−0.033	0.023	−0.078	0.011
Social Sciences × Age	−0.079**	0.030	−0.137	−0.020	− 0.100***	0.026	− 0.151	−0.049	− 0.014	0.019	− 0.052	0.023
Business × Age	−0.063	0.033	−0.127	0.002	−0.074**	0.022	−0.118	−0.030	− 0.001	0.021	− 0.042	0.041
Others × Age	−0.004	0.057	−0.116	0.108	−0.036	0.041	−0.117	0.045	0.017	0.040	−0.061	0.094
Multiple Fields^a^ × Age	−0.040	0.034	−0.107	0.026	−0.080	0.031	−0.140	−0.019	0.005	0.021	−0.035	0.046

*** *p <* .001, **p < .01, *p < .05

Abbreviations: *Ref.* reference, *S.E.* standard error, *C.I.* confidence interval

Sample includes only those individuals who attained a bachelor’s degree. All models control for aforementioned covariates

^a^Multiple Fields refers to individuals with a double major in different fields of college major only; individuals with a double major in the same field of college major are not categorized into Multiple Fields

Moving to the second and third columns, results from the subsample of men and the subsample of women show remarkable differences. Men who majored in STEM, social sciences, or business were associated with decreased substance use with age, compared to men in arts and humanities majors. However, for women, the rates of changes in substance use behaviors did not vary significantly by majors.

Finally, because it is possible that each substance use behavior may correlate with college major differently, we analyzed each substance use behavior separately (i.e., smoking, heavy alcohol use, and marijuana use). Table 5 shows the regression results from the multilevel models that estimated associations between college majors and each substance use behavior over time. We found that trends in heavy alcohol use and marijuana use were more likely to vary by college major than trends in smoking, which did not vary across college majors. For example, individuals who majored in the social sciences or business were associated with decreased heavy alcohol use with age, compared to individuals in arts and humanities majors. Additionally, individuals in social sciences or health majors were associated with decreased marijuana use as they aged.

**Table 5:** Longitudinal Results of the Association Between College Fields of Study and Substance Use Behaviors from Multilevel Logistic Regression (*N =* 1031)

	Smoking			Heavy Alcohol Use			Marijuana Use		
	O.R.	95% C.I.		O.R.	95% C.I.		O.R.	95% C.I.	
***Change in Health Risk Behaviors by Major***									
STEM × Age	1.026	0.844	1.248	0.935	0.796	1.097	1.036	0.852	1.260
Health × Age	1.150	0.829	1.596	0.790	0.620	1.005	0.704*	0.497	0.997
Education × Age	0.875	0.681	1.124	0.922	0.747	1.137	0.808	0.604	1.081
Social Sciences × Age	0.894	0.736	1.086	0.808*	0.685	0.952	0.781*	0.634	0.962
Business × Age	0.896	0.752	1.068	0.779**	0.666	0.910	0.965	0.804	1.158
Others × Age	0.797	0.548	1.160	0.993	0.729	1.354	1.061	0.750	1.502
Multiple Fields^a^ × Age	0.913	0.744	1.120	0.933	0.780	1.116	0.905	0.734	1.116

*** *p <* .001, **p < .01, *p < .05

Abbreviations: *Ref.* reference group, *S.E.* standard error, *C.I.* confidence interval, *O.R.* odds ratio

Note: Samples with attaining college degrees only. All models control for aforementioned covariates

^a^Multiple Fields refers to individuals with a double major in different fields of college major only; individuals with a double major in the same field of college major are not categorized into Multiple Fields

We also conducted a number of sensitivity analyses to check the robustness of the results. These analyses included: (1) investigating whether college major is associated with engaging in a substance use behavior in the past year (instead of the past 30 days) and (2) using multiple imputation instead of listwise deletion to recover missing values. The results from both sensitivity analyses were similar to the results in the main analysis. The results of all sensitivity analyses are available upon request.

## Discussion

The college years are a critical point in the life course when individuals build the foundation for a healthy and successful future. Whether college students develop healthy lifestyles and abstain from unhealthy substance use, particularly during the transition to adulthood, is thus a critical issue for public policy and practice. A student’s college experience is strongly shaped by his/her choice of major, with academic and social experiences differing greatly across majors. Nevertheless, the literature that seeks to understand college students’ health behaviors, including substance use, largely overlooks the role of college major. This question has gone unasked: Do all college degrees affect health behaviors in the same way, regardless of the field of study? This study sought to answer to this question by focusing on college major and substance use behaviors. Using longitudinal data from a national sample, the results revealed some interesting patterns between certain aspects of college education and substance use.

Consistent with prior studies, having a college degree was associated with a decreased likelihood of engaging in substance use behaviors (e.g., [2]). This study found some more specific variations in substance use by college major, particularly in terms of substance use prevalence and trajectories over time. Our analysis found that social science and business majors, on average, show higher rates of substance use than arts and humanities and STEM majors. Yet, social science and business majors also decreased their substance use more quickly over time than other majors did. When examining specific substance use behaviors, we found that college major is a significant predictor of heavy alcohol use and marijuana use, but not smoking. In addition, we observed strong gender differences: The associations we found between college major and substance use were more salient for men than women. Taken together, these findings lend some support to our hypothesis that college majors are heterogeneous and may potentially affect health behaviors, particularly substance use, differently.

The findings from this study are important because they inform the literature on college students’ health and substance use in several key ways. First, studies of substance use have traditionally focused only on the “social” and interpersonal” contexts. Indeed, the literature on adolescent health has long recognized the crucial role that peers and social context play in whether students engage in risky and unhealthy behaviors [51, 52], and some recent studies suggest that college students are no exception [53, 54]. However, particularly in college when the individual experience differs so much by major, it is also imperative to focus on a student’s ‘educational context’ and how it might relate to substance use and other health behaviors. By focusing on college major and substance use, this study demonstrates that important heterogeneity exists: Not all majors are associated equally with substance use behaviors and the patterns cannot be fully explained by standard social and interpersonal contexts, such as demographics, SES, employment, etc. Future studies can build on this research by digging deeper into college majors to elucidate the mechanisms through which they affect substance use behaviors or other health outcomes.

Second, the findings inform the literature by suggesting that business and social sciences majors may be less ‘healthy’, that is, that students in these majors have a higher likelihood of engaging in substance use behaviors than students in other majors. This finding deserves further discussion and, ultimately, further research. At first glance, the variations in substance use across majors may be assumed to be due to differences in health literacy, i.e., students in health-related majors are more aware of the health implications of their behaviors than other students. However, it is important to note that we found observable differences between students in arts and humanities and other, non-health fields of study; such differences cannot reasonably be explained by differences health literacy. For example, it is difficult to believe that arts and humanities majors would have better health knowledge than social science majors, such as political science, sociology, or pre-law [33, 55]. Also, the assumption that the differences are due to what a student’s major teaches about health literacy is of limited explanatory value when one considers that recent, widespread public health campaigns have given all young adults a more similar baseline of health literacy. In fact, a recent study finds no difference in terms of health literacy by college majors [56]. Unfortunately, the NLSY97 does not include questions about students’ health literacy during the college years, so the aforementioned hypothesis cannot be tested. We argue that the higher likelihood we observe among business and social science majors to engage in substance use behaviors is an empirical finding that must be explained, and that it is likely that factors such as the learning experiences and opportunities in the student’s major may help explain the differences. Lipson, et al. [57] provides support for this idea, suggesting that a highly competitive environment surrounded by peers and faculty in the arts and humanities may help explain the prevalence of students’ mental health problems. Mental health challenges, in turn, are often associated with an increase in risky and unhealthy behaviors. Future studies that specifically investigate the peer influence and substance use culture for heavy alcohol use, smoking, or marijuana use initiation across learning contexts by college major will yield valuable insights that refine our hypothesis.

Finally, and more broadly, the findings from this study suggest a conceptual reconsideration of the role of education in social epidemiology theory and research. Most social epidemiological research focuses on the health benefits of education, which have been largely understood to mean only educational attainment. Indeed, it has been well documented that college graduates are healthier and exhibit more healthy behaviors than those with less education [58–62]. But, the import of education is not limited to achievement, per se. College graduates differ from non-graduates because they have spent a substantial amount of time in educational settings. Yet, the literature has less to say about what experiences in that educational setting make a difference for later health behaviors. Our understanding of how a college student’s field of study (college major) impacts individual health remains very limited. Findings from this study demonstrate the heterogeneity of college majors with respect to substance use, and in so doing, suggest the need to expand the concept of education in social epidemiology research.

Despite the strength of this study, we recognize a few limitations. First, since students do not randomly select into their major, the results are not causal. There are factors related to students and their lives that affect the choice of major and the choice to engage in risky health behaviors. As such, even though we used longitudinal data and controlled for a wide range of potential confounders, our findings remain associational. In particular, there is some evidence that heavy drinkers appear to gravitate toward business majors, which makes the causal relationship even more complicated [63, 64]. Readers should be cautious and refrain from making causal interpretations of the associations reported in this study. Second, because the NLSY97 did not include measures of mental health in every wave of the survey, we could not control for mental health during the college years and thus test whether mental health mediates the associations we found. We hope that future research will shed light on this issue by examining mental health as it relates to college students’ field of study and substance use. Third, the definition of ‘heavy alcohol use’ used in the study differs slightly from the most-up-to-date definition of binge drinking, only because the NLSY97 survey question followed the earlier classification of binge drinking that was used in the National Survey on Drug Use and Health. As such, caution should be used when comparing our results on heavy alcohol use to other studies of binge drinking. Finally, despite the large sample size and detailed information on respondents’ college years, our findings might not be generalizable to current college students. The NLSY97 data is approximately a decade old, and college students today face a different policy context for substance use [65]. Future research that uses newer data may inform the generalizability of the NLSY97 data.

Limitations notwithstanding, this study demonstrates that even among people who have all completed college, there are significant variations in engaging in substance use, an important health risk behavior. The associations cannot be explained by demographic characteristics, familial SES background, and respondents’ employment and economic well-being. This robust conclusion has three practical implications. First, college health centers need to work with deans and department chairs in fields with a higher risk of substance use (i.e., business, social sciences) to increase awareness of the issue. Going further, our results should encourage deans and department chairs to consider not just the academic preparation of their majors for career success in terms of job placement and salary [66], but also the preparation of their majors for life success in terms of health behaviors. Our study suggests some majors come with a higher lifelong price, i.e., higher health risk. Increasing awareness of this issue is the very first step. Second, college health centers need to play a more active role in building a healthy culture among faculty members and students in fields that are more vulnerable to substance use. The idea that college major may affect substance use behaviors can be used to promote more collaboration between school health centers and leaders in academic divisions and departments. Finally, our findings call for deans and department chairs to pay greater attention to how the distinct learning and professionalization environment that their major provides might impact future health behaviors. In short, health should become a consideration in future curriculum design.

## Conclusions

This study used a large-scale, longitudinal dataset to investigate whether and how engagement in substance use behaviors varies by college major. We find that not all college majors show the same level of engagement in substance use behaviors over time, and that the associations also vary by the specific substance use behavior examined and by gender. These findings suggest it is important to consider that the different learning and educational contexts that college majors provide may also be more or less supportive of substance use behaviors. Going further, our findings should higher education administrators to consider not just the academic preparation of different college majors for career success in terms of job placement and salary, but also the preparation of different majors for life success in terms of health behaviors. Ultimately, findings of this study may promote a more comprehensive understanding of the educational context of college, and how it affects not only students’ learning but also their substance use, will help us better prepare college students for career and life success.

## Data Availability

The authors have full access to the NLSY97 public-use dataset. Data can be downloaded from the following link: https://www.nlsinfo.org/investigator. The analytical sample and codes used and/or analyzed during the current study are available from the corresponding author, on reasonable request.

## Appendix

**Table 6:** Pair-wise Comparisons of Change in Substance Use Behaviors Across College Fields of Study from Longitudinal Multilevel Negative Binomial Regressions

	Arts & Humanities	STEM	Health	Education	Social Sciences	Business	Others	Multiple Fields^a^
Arts & Humanities								
STEM	–							
Health	–	–						
Education	–	–	–					
Social Sciences	A&H > SS	STEM > SS	–	–				
Business	A&H > B	–	–	–	–			
Others	–	–	–	–	–	–		
Multiple Fields^a^	–	–	–	–	–	–	–	

Abbreviations: *A&H* Arts & Humanities, *SS* Social Sciences, *B* Business

Samples with attaining college degrees only; Significant difference (*p <* .05) presented only

^a^Multiple Fields refers to individuals with a double major in different fields of college major only; individuals with a double major in the same field of college major are not categorized into Multiple Fields
